# The Study of Influence of Sound on Visual ERP-Based Brain Computer Interface

**DOI:** 10.3390/s20041203

**Published:** 2020-02-21

**Authors:** Guizhi Xu, Yuwei Wu, Mengfan Li

**Affiliations:** 1 State Key Laboratory of Reliability and Intelligence of Electrical Equipment, Hebei University of Technology, Tianjin 300132, China; gzxu@hebut.edu.cn (G.X.); 201821402010@stu.hebut.edu.cn (Y.W.); 2 Tianjin Key Laboratory of Bioelectromagnetic Technology and Intelligent Health, Hebei University of Technology, Tianjin 300132, China

**Keywords:** brain–computer interfaces (BCI), event-related potential (ERP), visual stimulus, auditory task, mental workload

## Abstract

The performance of the event-related potential (ERP)-based brain–computer interface (BCI) declines when applying it into the real environment, which limits the generality of the BCI. The sound is a common noise in daily life, and whether it has influence on this decline is unknown. This study designs a visual-auditory BCI task that requires the subject to focus on the visual interface to output commands and simultaneously count number according to an auditory story. The story is played at three speeds to cause different workloads. Data collected under the same or different workloads are used to train and test classifiers. The results show that when the speed of playing the story increases, the amplitudes of P300 and N200 potentials decrease by 0.86 μV (*p* = 0.0239) and 0.69 μV (*p* = 0.0158) in occipital-parietal area, leading to a 5.95% decline (*p* = 0.0101) of accuracy and 9.53 bits/min decline (*p* = 0.0416) of information transfer rate. The classifier that is trained by the high workload data achieves higher accuracy than the one trained by the low workload if using the high workload data to test the performance. The result indicates that the sound could affect the visual ERP-BCI by increasing the workload. The large similarity of the training data and testing data is as important as the amplitudes of the ERP on obtaining high performance, which gives us an insight on how make to the ERP-BCI generalized.

## 1. Introduction

The brain–computer interface (BCI) is a system to control the machine without human peripheral neuromuscular system [1,2]. This can effectively enhance the physical ability of the user [3,4,5]. Event-related potential (ERP) is an evoked potential recorded from the surface of the scalp when a person performs cognitive processing (e.g., attention, memory, thinking) with a particular stimulus, which reflects the neurophysiological changes of brain during cognitive processes [6,7]. Less training and high performance make the ERP-based BCI system widely used, e.g., underwater manipulator [8], consciousness detection [9], paradigm research [10,11].

The ERP-based BCI meets a common problem: the performance of the BCI decreases when applying it into the real environment [12,13]. The sound widely exists in the daily life. When the subject uses the BCI to output commands, he/she is unavoidably affected by the sound [14], such as talking voice, robot walking step, the sound of the machine, and so on. Whether the sound affects the performance of the visual ERP-based BCI is unclear, since there are two different views on the influence of the sound. On one hand, some researchers have shown that using the visual and auditory tasks simultaneously could improve the performance [15] since the redundancy effect makes the subject use less time to react [16]. On the other hand, the auditory pathway has an inhibitory effect on the visual pathway [17], which leads to the attention paid on the visual stimulus being affected by the sound [18]. Therefore, it is necessary to analyze the effect of the sound to find the main reason for causing the performance decline in this study.

How to evaluate the effect of the sound is also a challenge. Table 1 lists some studies on the influence of the sound in the terms of emotion [19,20], stimulus-onset asynchrony [21], natural sound, and music [22,23,24]. Akinari, O. et al. found the sound with different emotions to have an influence on the auditory BCI. The auditory BCI under a very negative sound can achieve 84.1% classification accuracy [19]. Affective stimuli may be useful for a practical auditory BCI system for patients with disabilities, which achieve 90% classification accuracy [20]. Sijie, Z. et al. reported that the background music does not impair the auditory BCI but is preferred by users [22]. Baykara E. et al. found training and enhanced motivation improves performance in an auditory BCI paradigm [23]. We predict that less motivation will reduce the performance in visual BCI. Eva M.H. et al. discovered many psychological factors which improve the performance auditory BCI and visual BCI [24]. Jeong H. et al. found that an auditory steady-steady-state response (ASSR) BCI under the music and natural sounds achieves 89.67% and 87.67% classification accuracy because of the acceptance of user increasing [25]. Hirotaka N. et al. used the support vector machine to improve the performance of ASSR-BCI under an additive noise condition, which was improved by 10.5% compared to conventional ASSR-BCI under noiseless condition [26]. First, most researches explore the influence of sound to auditory BCI. They are in a single pathway. We explore the influence that the sound has on the visual BCI in a dual pathway. Second, this study mainly focuses on whether the speed of the sound can affect the BCI since the speed change can directly influence the amount of information processed by the brain, which causes the change of the workload assigned to the BCI task. We use an auditory task that is with higher speed to reduce the allocation of brain resources for the visual BCI. In our work, we reduced the motivation in the use of visual BCI through a speed auditory task.

Mental workload is the ratio between the brain resources required by the task and the available brain resources of the operator [27,28]. Since the visual BCI requires the subject to be with a suitable mental workload [29] to reach a high performance, this study assumes that the sound might influence the BCI performance by having an effect on the mental workload. This study proposes to use the speed of sound to adjust the mental workload. The speed of sound represents the number of words played per minute, which affects the amount of information received by the brain. The increase of information input would increase the load of processing them in the brain, which might reduce the efficiency of dealing with the BCI task, since the limited workload of the brain cannot handle the sound with high speed and BCI task simultaneously. Compared with the researches studying the auditory BCI, the novelty of this study is to propose a dual-task that includes both the visual BCI task and auditory task. The dual-task has advantages in activating both the visual and auditory pathways in the brain and is helpful for analyzing the interaction of the two pathways when the subject is under a BCI system.

It is a problem whether the speed auditory task decreases the performance of the visual BCI when the speed of auditory task occupies more brain resources. And whether the auditory task has an inhibitory effect on the visual pathway is also a problem. In order to test the effect of the sound on the visual ERP-based BCI, this study designs a dual-task experiment that requires the subject to do the visual BCI task and auditory task simultaneously. The auditory tasks are designed as three levels to simulate the daily cases in which the subject is affected by the sound in different indexes. National Aeronautics and Space Administration-Task Load Index (NASA-TLX) is used to indicate the subjective index of the workloads. We evaluate the effect by the amplitude, accuracy rate, and information transfer rate. In addition, we use the data collected under the same or the different workloads to train and test the classifier. The results show that the amplitudes of N200 and P300 potentials decreased from 2.59 μV to 1.90 μV and from 3.20 μV to 2.43 μV in occipital area when the workload increased. The results indicate that auditory noise has interference in the occipital area when inducing ERP. High workload also leads to low accuracy and information transfer rate. The accuracy and information transfer rate (ITR) of the high workload are 90.83% and 27.68 bits/min, which are higher than the low workload. After the experiment, we found that as the brain resource becomes less, the visually-induced ERP is reduced. The decrease of the ERP amplitude makes the classification accuracy and ITR decrease, which causes the visual BCI performance to decline. Besides, a large difference between the training data and testing data also leads to a decrease of the performance. These findings imply that the auditory task could make the N200 and P300 potentials different from the training phase to the testing phase by increasing the subject’s workload, which influences the visual ERP-based BCI performance. This finding is helpful for further designing a more robust visual ERP-based BCI.

Section 1 is the introduction that describes the reason why ERP-BCI is used widely and the existing problems in ERP-BCI. Section 2 introduces the design of the experiment and the index of evaluation. Section 3 describes the comparison of subjective fatigue values and the ERP amplitudes, as well as the accuracy and information transmission rates. Section 4 discusses the factor why these values have differences in different auditory tasks. Finally, a brief conclusion is given in Section 5.

## 2. Materials and Methods

### 2.1. Participants and Data Collection

Ten subjects (nine males) from the Hebei University of Technology with a mean age of 22 years (range from 19 to 24) participated in the electroencephalography (EEG) session of this study. All participants had normal or corrected-to-normal vision and were informed about the risk of seizures in epileptics due to flicker stimulation. They reported not to have ever suffered from epilepsy and gave their written informed consent. One was familiar with this experiment, and the rest had no experience. All subjects gave their informed consent for inclusion before they participated in the study. The study was conducted in accordance with the Declaration of Helsinki, and the protocol was approved by the ethics committee of the Hebei University of Technology (HEBOThMEC2019001).

All electrode impedances were reduced to 10 kΩ before data recording. EEG signals were sampled at 1000 Hz (SynAmps2, Neuroscan, USA). All 36 channels were grounded between Fpz and Fz channel and referenced to the binaural mastoid.

### 2.2. Visual Interface

**Figure 1:**
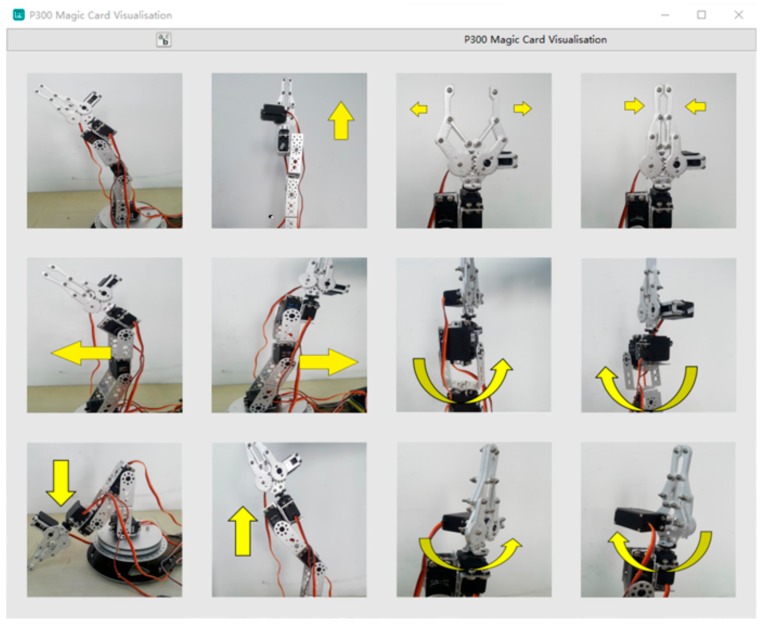
Stimuli interface.

Figure 1 shows the interface and protocol developed in the OpenViBE environment. There are 12 robotic static images in the stimulus interface, which is a 3 × 4 matrix. Each image represents a robotic arm behavior. To encode robotic arm behavior, we define row *i* and column *j* in the matrix as location (*i*, *j*), as shown in Table 2. We use this interface to activate the visual stimuli. In this oddball paradigm-based interface [30], visual images flash randomly one by one. When a visual image is presented, a black square with a white solid circle shields the others. The image that the subject focuses on is the “target”, and the image ignored is defined as “non-target”. At the beginning of the experiment, the subjects watch the interface. Twelve pictures flash randomly, and the time each picture flashes is 150 ms, pausing for 75 ms after the flashing; the remaining 11 pictures flash randomly. The stimulus onset asynchrony is set to 225 ms. A process in which the interface flashes each stimulus once is defined as a “repetition”. In our study, 10 repetitions constitute a “trial”, and the target in the trial stays the same while the other stimuli are non-targets; the interval of every trial is 500 ms. One experiment session consists of 12 trials, and each subject conducts three sessions in this study.

### 2.3. Visual-Auditory BCI Tasks

The auditory material is an excerpt of audio story “Like a Flowing River”. The time length of the material is 8 min.

**Figure 2:**
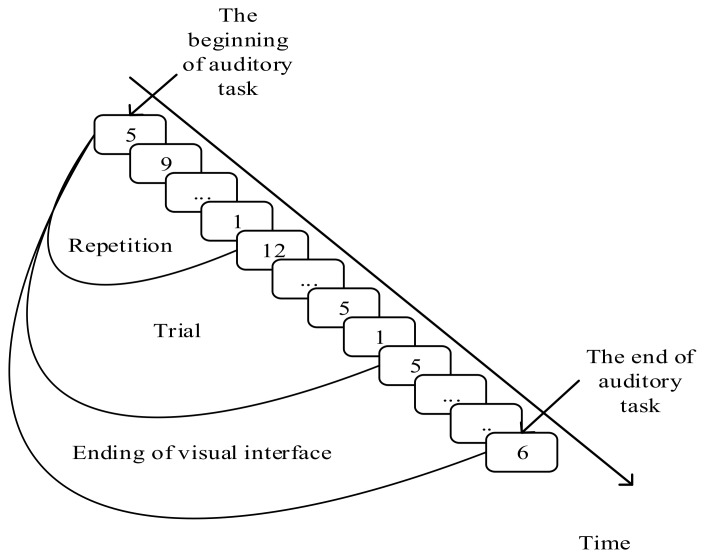
Timing diagram of visual-auditory task.

The visual-auditory BCI tasks are categorized into three types. In each type, the subjects need to conduct 36 trials. Every trial is composed of 10 repetitions. Every 12 trials consist of a sub-experiment in which a certain auditory material is played when the sub-experiment starts and is stopped when the sub-experiment ends. In each sub-experiment, the subject is required to not only focus on the target stimulus in each trial, but also silently count the number of “De” noticed from the auditory material. The differences between the three tasks are the speeds of playing the material: 0–auditory task (0-T) represents the task in which there is no auditory material played; 0.5–auditory task (0.5-T) represents the task in which the auditory material is played in half of the ordinary speed; 1–auditory task (1-T) represents the task in which the auditory material is played in an ordinary speed. The numbers of the “De” in the three tasks are 0, 26 and 53. The procedure of the visual-auditory BCI tasks is shown in Figure 2.

### 2.4. The NASA-TLX Scale

**Figure 3:**
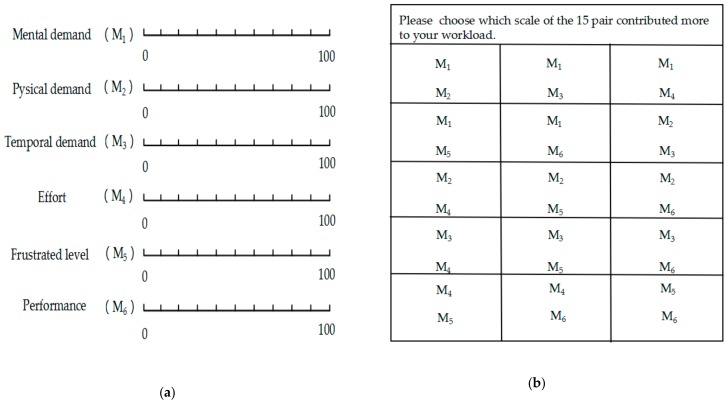
The fatigue value obtains from the scale of NASA-TLX: (**a**) The six aspects; (**b**) The fifteen pairs regarding the six aspects.

Subjects complete the fatigue scale after the experiment, and the average value the subjects get from the scale can determine the difficulty of the auditory task; the difficulty has three levels: low, medium and high. The most commonly used subjective fatigue scale is the NASA-TLX scale developed by NASA, which has good internal consistency and structural validity as a subjective fatigue assessment for BCI system operations [27]. In Figure 3a, we can get the scores of M_1_ to M_6_. In Figure 3b, we can calculate N_1_ to N_6_ from the 15 pairs comparisons. The larger the total score, the greater the mental workload.

Its score expression is:

(1) $$F=\sum_{i=1}^{6}\frac{Ni}{C_{6}^{2}}\times M_{i}$$

where *F* is the mental workload value of the subject, and $N_{i}$ is the number of *i*-th dimensions that participants considered to have more contribution to the overall mental workload than other dimensions. The $C_{6}^{2}$ means the number of pair-wise comparisons of all factors. $M_{i}$ is the score of the *i*-th dimension.

### 2.5. Feature Extraction and Classification

The EEG data from 0 ms to 600 ms post-stimulus is cut for each stimulus. Firstly, a 3rd-order Butterworth band-pass filter (0.5-10 Hz) is used to filter each epoch. Secondly, the data of 50 ms pre-stimulus is used as the baseline to make a baseline correction. Thirdly, all epochs are down-sampled to 40 Hz to reduce the data size. At last, this experiment uses 36 channels as feature channels to output a 864-dimension feature vector.

The fisher linear discriminant analysis (FLDA) is used for the binary classification problem because they have excellent classification performance in ERP-based BCI systems to solve identification problems [31,32].

The discriminant function of the Fisher classifier is:

(2) $$y=w^{T}x+\omega_{0}$$

$w^{T}$ is the best projection direction, x is the data sample, and $\omega_{0}$ is the central value of the two types of data; the samples are classified by the value of y: the samples is discriminated as a target when y> 0 and as a non-target when y< 0.

The mental workload of different people is affected by many factors, so the fatigue of each subject after an auditory task has three states which are low workload state (LD), medium workload state (MD) and high workload state (HD). The rule of division is as follows:

(3) $$\left\{\begin{array}{l} V_{j}\geq V_{m}\wedge V_{j}=\min\left\{V_{j-0},-,V_{j-0.5},V_{j-1}\right\},\;V_{j}\in LD\\ V_{j}\geq V_{m}\wedge V_{j}=\max\left\{V_{j-0},-,V_{j-0.5},V_{j-1}\right\},\;V_{j}\in HD\\ else,\;V_{j}\in MD \end{array}\right.$$

where *V_j_* is the fatigue value of the *j*-th subject after completing the visual-auditory BCI task. *V_i_*_-0_, *V_i_*_−0.5_, and *V_i_*_−1_ are the fatigue values of 0-T, 0.5-T and 1-T visual-auditory BCI tasks.

To determine the effects of auditory task on the performance of the BCI, nine train-test combinations are created, namely L-L, L-M, L-H, M-L, M-M, M-H, H-L, H-M, and H-H. The first three combinations, L-L, L-M, and L-H, represent the conditions in which BCI are built under the LD condition and tested separately under LD, MD and HD. M-L, M-M, and M-H represent the conditions in which BCI are built under the MD condition and tested separately under LD, MD, and HD. The last three combinations, H-L, H-M, and H-H, represent the condition in which BCI are separately trained on HD and tested on LD, MD, and HD.

### 2.6. Evaluation Criteria

The classification accuracy in this article refers to the ratio of the number of correctly classified “Trial” and the total number of “Trial”.

(4) $$P=\frac{T_{r}}{T_{t}}\times 100\%$$

where P is the accuracy, $T_{r}$ is the correctly classified number of “Trial”, and $T_{t}$ is the total number of “Trial”, which is 36 in this research.

The information transfer rate (ITR) was originally used for the communication and computational rates of measurement systems in the communications field and was introduced in the BCI field by the Wolpaw et al. [32,33]. Due to the three basic performance indicators of optional target number, target recognition accuracy, and single target selection time, ITR has become one of the most commonly used comprehensive evaluation indicators for evaluating system communication rates in BCI research. Before calculating the ITR, we first need to calculate the amount of information transmitted by a single target selection, that is the bitrate (*B*). The formula of *B* is:

(5) $$B=\log_{2}N+P\log_{2}P+(1-P)\log_{2}(\frac{1-P}{N-1})$$

The unit of *B* is bits/selection, the *N* represents the number of possible targets (12 with the 3 × 4 matrix), and the *P* represents the accuracy that are correctly classified (average accuracy).

The formula of ITR is:

(6) $$\mathrm{ITR}=B\times \frac{60\times 1000}{n\times t_{r}+t_{i}}$$

The *n* represents the repetition number of trials, the $t_{r}$ represents the time of a repetition which is 2700 ms, the $t_{i}$ represents the interval time between each trial, which is 500 ms.

## 3. Results

### 3.1. Behavior Data

From Table 3, the average number of “De” that 10 participants count is close to the actual number in the auditory story. The average recognition error of all the participants fluctuates within an acceptable range. It can be considered that the subjects completed the auditory task during the experiment. All the participants completed the auditory tasks well, indicating that the difficulty of the auditory tasks is designed reasonably. In addition, as the difficulty of the auditory task increases, the error fluctuation increases from 1 to 3 on average. This indicates that high-speech auditory tasks will increase the probability of misidentification, and this auditory task is more difficult. Additional cognitive tasks are more difficult, and the speed of auditory material is the factor that affects the difficulty of the task itself. We cross verify the subjective reports by using Ura’s variation of Scheffe’s method [20]. Table 3 shows the difficulty of each sound. The rated difficulty is analyzed by the analysis of variance that contains factors of the average of ratings, the individual difference of the ratings and so on. It reveals that the significant main effects come from the average of ratings (*p* < 0.01).

The fatigue values of three auditory tasks about all subjects are provided in Table 4. We use the mean as the fatigue value of the three auditory tasks [34,35,36]. The fatigue values of 0-T, 0.5-T and 1-T are 34.98, 50.09 and 54.87. According to the fatigue values of three auditory tasks, the auditory task 0-T is defined as low difficulty task, 0.5-T is defined as medium difficulty task (*p* < 0.05, *p* = 0.0282), and 1-T is defined as high difficulty task (*p* < 0.05, *p =* 0.0073). The behavioral results (the error number of the “De”) and the NASA-TLX scale scores prove that controlling the difficulty of the auditory task is achieved. The mental workload of the subjects in the process of performing the BCI task has reached different status. We can conclude that the subjects can produce a stronger fatigue response after experiencing a faster speaking rate auditory task. This reaction can be seen from the change of fatigue scale values. Different subjects think the auditory task is different in difficulty. The fatigue values of S2, S6 and S8 do not decrease with the increased difficulty of the auditory task.

The subjects’ different fatigue values imply that individual differences also exists in the subjects’ feeling on the tasks. The addition of auditory task to the subject’s visual task while getting stimulated also induces ERP. In Section 2, we defined the LD, MD and HD according to the fatigue value that is calculated after the subject makes the experiment, so we take the EEG data of three statuses after the experiment to analyze.

### 3.2. The Analysis of ERP

**Figure 4:**
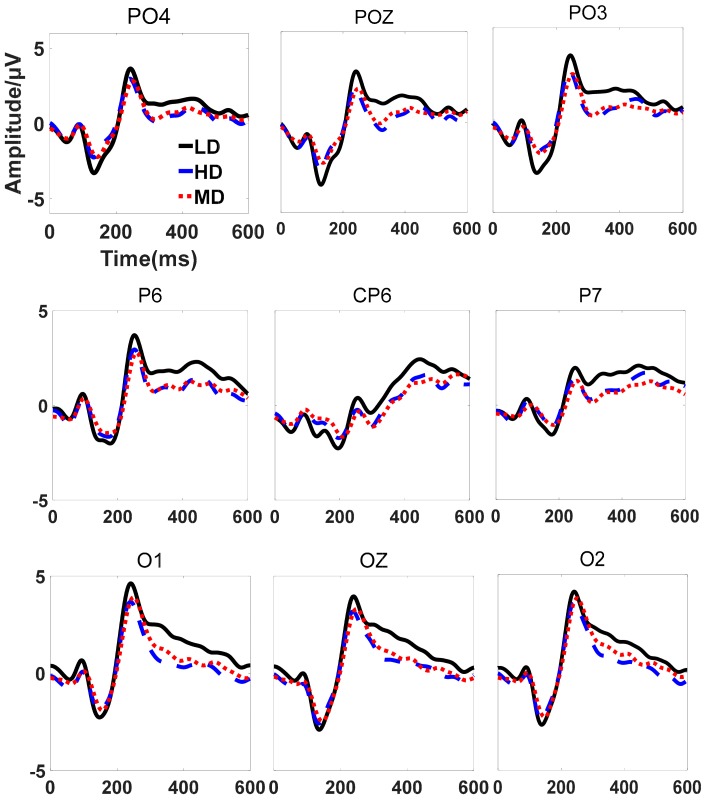
The comparison of ERP amplitude changes of some channels under different mental workload states.

The off-line analysis extracted features of the ERP. Brain signals within a 600 ms window, from 0 ms pre-stimulus to 600 ms post-stimulus, were selected and pre-processed using the following steps. First, a digital filter with a bandwidth of 0.5–10 Hz filtered the signals. Second, a baseline corrected the signals by subtracting the mean value of the data from 300 ms pre-stimulus to the time point when the stimulus appeared. Finally, an algorithm averaged the epochs induced by the same type of stimuli (target or nontarget) to extract the ERP’s patterns. The grand averaged ERP waveform for PO4, POZ, PO3, P6, CP6, P7, O1, OZ, and O2 are depicted in Figure 4. The common ERP components for oddball paradigms like N200 and P300 can be observed from the waveform. In Figure 4, the ordinate represents the magnitude of the ERP amplitude. The abscissa is the time after the target stimulation. The black solid line represents the amplitude changes under the LD. The blue dashed line represents the amplitude change under the MD, and the red dashed line represents the amplitude change under the HD. The latency and amplitude of some channels is shown in Table 5. The latency of N200 and P300 under higher workload state is delayed about 6 ms compared to lower workload state. The P300 amplitude of parietal and occipital areas decreased with the increase of additional auditory difficulty tasks. The possible reason may be that working memory occupies the mental resources that generate P300.

The average amplitude of P300 decreased by 0.74 μV (*p* = 0.0104) under MD compared with LD, and decreased by 0.12 μV (*p* = 0.0489) under HD compared with MD in occipital area. The average amplitude of N200 decreased by 0.56 μV (*p* = 0.0055) under MD compared with LD, and the N200 amplitude induced decreased by 0.13 μV (*p* = 0.0383) under HD compared with MD in the occipital area. In addition, the amplitude of N200 in central and parietal areas also decreased with the increase of additional auditory difficulty tasks. The changes of N200 and P300 amplitude between MD and LD have more obvious differences than that between HD and MD. Both the amplitudes of N200 and P300 in parietal area decrease with the workload state increase. The P300 amplitude in parietal area has the fastest decline under MD compared with LD, and the N200 amplitude in the parietal area has the fastest decline. This indicates that the higher workload the subject has, the lower the ERP amplitude in occipital and parietal areas. The amplitude of the P300 of occipital area in HD is decreased by 0.86 μV (*p* = 0.0239) compared to the LD, which indicates the subjective and objective data are consistent.

**Figure 5:**
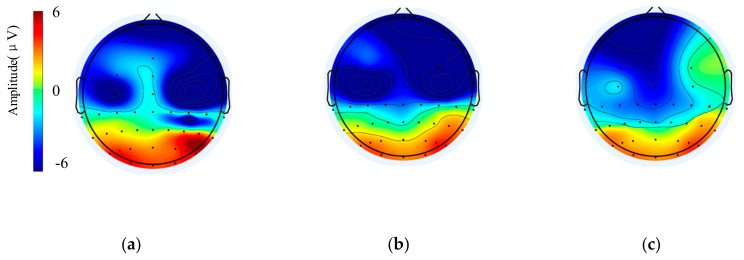
The topographic map of the brain under different mental workloads: (**a**) 250 ms after stimulation under LD; (**b**) 250 ms after stimulation under MD; (**c**) 250 ms after stimulation under HD.

Next, we observe the changes in the region of the brain related to the vision from the brain topographic map. Figure 5 shows patterns of brain activities induced by the target stimulus at the post-stimulus time of 250 ms under LD, MD and HD. The colors of the topographies represent the amplitudes of the 10 subjects’ average brain signals. The scale in Figure 5 is 6 μV. The amplitude in the occipital region of the brain increase at 250 ms after receiving the visual stimulus. When the subject is in a high mental workload state, the amplitude of the occipital area gradually becomes the least.

In Figure 5, the amplitude under LD in the occipital area is the maximum, MD is next, and the amplitude of the occipital area under HD is the minimum. The topographic map changes corresponding to the amplitude changes in Table 4. It has the most obvious amplitude decrease in the occipital region of the brain, with the increase of the mental workload of the subject. This indicates that when the additional auditory tasks become more difficult, the brain is in a higher fatigue state, and the magnitudes of N200 and P300 in the occipital region of brain decrease.

### 3.3. Accuracy and ITR

**Figure 6:**
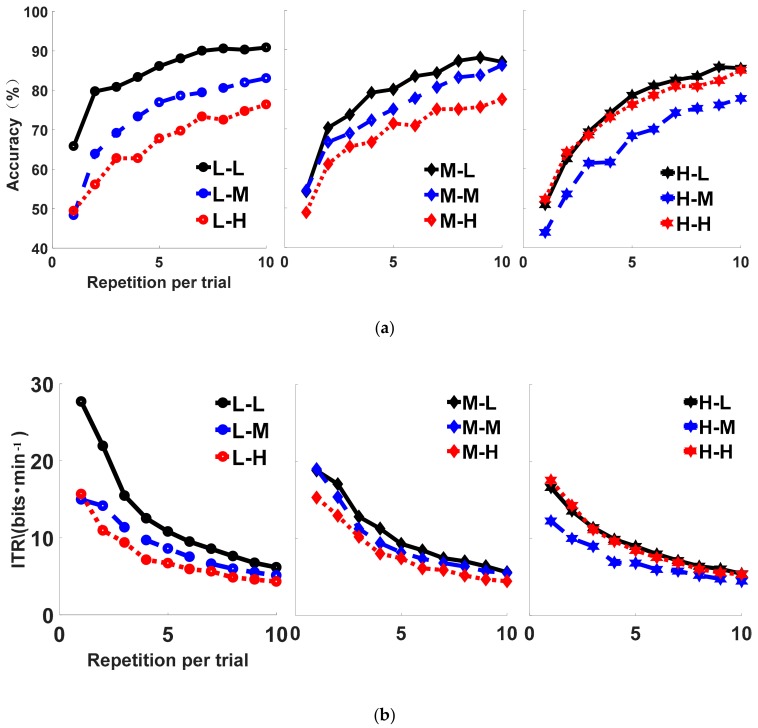
The changes of accuracy and ITR under different mental workloads: (**a**) The changes of accuracy under different mental workloads; (**b**) The changes of ITR under different mental workloads.

The average classification accuracy and the ITR of all subjects are in Figure 6, where the abscissa represents the repetition number of a Trial. The ordinate represents the classification accuracy and the ITR respectively. The same color of line indicates the same test set. The same mark of line indicates the same train set. As the number of repetitions increases, the classification accuracy increases and the ITR decreases. After 10 repetitions, the curves L-L (90.83%, 5.65 bits/min), M-L (86.94%, 5.15 bits/min) and H-L (85.41%, 4.96 bits/min) are almost the highest, which means the accuracy and ITR reach the highest when using the data of LD as the test set. In Figure 6, L-L (90.83%, 16.43 bits/min) and M-L (86.94%, 5.15 bits/min) are the highest, followed by L-M (83.05%, 4.68 bits/min) and M-M (86.11%, 5.05 bits/min), and L-H (76.39%, 3.96 bits/min) and M-H (77.49%, 4.07 bits/min) are the lowest. This indicates that the classification accuracy is the highest with the data of LD for testing, followed by the data of MD for testing, and the lowest with the data of HD for testing except using the data of HD as the training set.

## 4. Discussions

Many BCI systems perform well in the lab environment, but the performance becomes poor when applying it into the real environment. It is necessary to find the reason that causes this decline to retain a high performance. Since there are many potential factors influencing the BCI performance in the real environment and the sound is a common noise, this study mainly analyzes the influence of the sound.

### 4.1. The Auditory Task Increases the Mental Workload

We can see that most of the subjects regard the task with higher speed as harder. Higher speed makes the subjects receive and deal with more information within unit time, which causes an increase of mental workload. When the amount of information is too much, the subjects might ignore some information, which leads to decision mistakes, such as counting the number of “De” wrongly, or forgetting to pay attention to the target stimulus when it appears. This can be seen from the behavior results, where as the speed increases, the rate of counting correctly falls.

On the other hand, as the difficulty increases, the ERP decreases. The average amplitude of P300 is decreased by 0.86 μV under HD compared with LD in occipital area (*p* = 0.0239). The average amplitude of N200 is decreased by 0.69 μV under HD compared with LD in occipital area (*p* = 0.0158). The subjects allocate less resources to the visual stimulus because he/she pays parts of attentions to the auditory task, which leads to lower ERPs when stimulated by the stimulus.

### 4.2. The Factors of Performance Decline

When applying the BCI into the environment where the speed of the sound is higher, the performance of it declines. The accuracy or the ITR in the LD conditions are all higher than the accuracy or ITR of the HD (MD & LD: *p* = 0.0282; HD & LD: *p* = 0.0073) conditions. The accuracy is also decreased by 5.95% in HD compared with LD (*p* = 0.0101). Finding the reason is important for using the BCI in daily life. This study uses the data sets under the same or different data sets to explore the factors influencing the detection performance. The nine conditions can be categorized as two types: The type that uses the same data sets to train and test the classifier, such as L-L, M-M, and H-H; and the type that uses the different data sets to train and test the classifier, such as L-M, L-H, M-L, M-H, H-L, and H-M. We assume that the performance of the classification in the BCI is influenced by two factors.

Firstly, the amplitude of the ERP has an influence on the performance. In Figure 6, as the speed of the auditory task increases, the performance of the classifiers of the first type decreases. The descending order of the three classifiers is L-L, M-M, H-H. This order is in accordance with the order of the amplitude under different tasks. Since the only difference between these classifiers is the amplitude, the amplitude affects classification. Higher amplitude could produce more obvious features, which leads to higher classification accuracy rate.

Secondly, the difference between training and testing data sets plays important roles on the performance. When using the same data to test the classifiers that are trained with different data sets, the results show that the L-L outperforms the M-L and H-L, the M-M outperforms the L-M and H-M, and the H-H outperforms the M-H and L-H. The accuracy and ITR under H-H are higher than the accuracy and ITR under H-M; the accuracy and ITR are the highest under L-L. The ITR is decreased from 16.43 bits/min under H-H to 12.36 bits/min under H-M. This indicates that the classifier performs best if the training and testing data are from the same task, even though the amplitude is not the highest. This finding indicates that if the testing environment is too noisy, the training process should take the noise into consideration to obtain better performance. When the training set data is inconsistent with the test set data, the classification accuracy and information transmission rate will decrease. When the subjects are in a low brain workload, or the classifier is trained and tested using the same state data, the classifier can obtain better classification and information transmission rate.

## 5. Conclusions

This study uses a dual-task to explore the influence of the speed of the sound on the visual BCI. The P300 and N200 both decline by 0.86 μV (*p* = 0.0239) and 0.69 μV (*p* = 0.0158) when the speed of the sound increases, which leads to a substantial decrease of accuracy (5.95%, *p* = 0.0101) and ITR (9.53 bits/min, *p* = 0.0416) of the visual BCI. The result demonstrates that increasing the speed of the sound has an effect on the allocation of the workload for the visual BCI task, which indicates that the auditory and visual pathways are related when dealing with the BCI. Besides, we find that the performance of the BCI also depends on the difference between the training and testing data. Large differences would lead to bad performance even though the classifier is trained well under the training environment; therefore, we should take the noise of the testing environment into consideration when training a classier.

The subject could hear the sound of servos of the robotic arm when conducting the on-line experiments. Whether this sound has an effect on the BCI performance needs to be studied in our future work. In addition, the visual BCI should take more kinds of visual images, such as the subject’s arm, to explore the way of improving the BCI performance. Whether the user’s familiarity with the sound or the memory cognitive mechanism of the human will influence the visual BCI performance needs to be studied in our future work.

## Figures and Tables

**Table 1:** Lists of studies on influence of sound on BCI.

The Literature	Electroencephalography (EEG) Method	Single Pathway (Y/N)	Dual Pathway (Y/N)	Factors of Influencing the Brain–Computer Interface (BCI) (Unrelated Possibility of Influencing the BCI)	Accuracy (%)	Performance (↑/↓)
[19,20]	ERP	Y	N	Positive or negative sound	84.1–90.0	↑
[21]	ERP	N	Y	Difference of the cognitive process in the brain	/	↑
[22]	ERP	Y	N	(Background music)	74.2	↑
[23]	ERP	Y	N	Training and motivation	>70.0	↑
[25]	ASSR	Y	N	Music and natural sounds	87.67–89.67	↑
[26]	ASSR	Y	N	Random noise	77.2	↑

**Table 2:** The content of stimulation image.

Picture Number	Robotic Status	Location
1	Recovery	loc (1, 1)
2	Upright	loc (1, 2)
3	Open	loc (1, 3)
4	Close	loc (1, 4)
5	Forward	loc (2, 1)
6	Backward	loc (2, 2)
7	Clockwise rotation	loc (2, 3)
8	Anticlockwise rotation	loc (2, 4)
9	Down	loc (3, 1)
10	Up	loc (3, 2)
11	Arm clockwise rotation	loc (3, 3)
12	Arm anticlockwise rotation	loc (3, 4)

**Table 3:** The number of “De” the subjects count.

Subject	Auditory Task		
	0-T	0.5-T	1-T
S1	0	26	53
S2	0	30	56
S3	0	25	44
S4	0	20	46
S5	0	25	51
S6	0	31	46
S7	0	27	48
S8	0	21	49
S9	0	21	49
S10	0	26	53
Mean	0	25	50

**Table 4:** Average fatigue value.

Subject	Auditory Task			Average
	0-T	0.5-T	1-T	
S1	35.78	59.78	67.56	54.37
S2	28.89	63.78	56.67	49.78
S3	42.89	76.89	84.44	68.07
S4	27.33	46.44	49.78	41.19
S5	19.33	24.00	30.67	24.67
S6	28.89	43.11	40.89	37.63
S7	24.00	31.78	42.00	32.59
S8	58.22	55.56	64.00	59.26
S9	37.33	47.33	48.89	44.52
S10	47.11	52.22	63.78	54.37
Mean	34.98	50.09	54.87	

**Table 5:** Latency and amplitude changes of some channels.

Channel	Latency(ms)						Amplitude(µV)					
	P300			N200			P300			N200		
	LD	MD	HD	LD	MD	HD	LD	MD	HD	LD	MD	HD
CP6	255	254	260	195	197	208	0.39	-0.23	-0.32	-2.27	-1.73	-1.66
O2	242	242	251	139	138	148	4.14	3.55	3.79	-2.68	-2.39	-2.28
Oz	239	238	248	136	134	145	3.95	3.18	3.31	-2.90	-2.58	-2.46
P6	251	251	258	176	172	160	3.79	2.95	2.77	-2.02	-1.66	-1.46
POZ	242	238	249	129	126	136	2.81	1.83	1.85	-3.46	-2.56	-2.25
P7	251	250	256	177	178	182	1.98	1.34	1.29	-1.56	-1.14	-1.05
PO3	244	242	251	138	138	150	3.50	2.59	2.49	-2.87	-1.76	-1.81
PO4	244	242	250	133	133	142	3.64	2.96	2.81	-3.31	-2.26	-2.23
O1	241	241	249	147	147	156	4.63	3.68	3.91	-2.27	-1.87	-1.91
